# CRISPR-Cas Gene Editing Technology in Potato

**DOI:** 10.3390/ijms26157496

**Published:** 2025-08-03

**Authors:** Zagipa Sapakhova, Rakhim Kanat, Khanylbek Choi, Dias Daurov, Ainash Daurova, Kabyl Zhambakin, Malika Shamekova

**Affiliations:** 1 Institute of Plant Biology and Biotechnology, Almaty 050040, Kazakhstan; z.sapakhova@ipbb.kz (Z.S.);; 2 Tanir Research Laboratory, Almaty 050060, Kazakhstan

**Keywords:** potato, gene editing, CRISPR-Cas, improve breeding process, biotic stress, disease resistance, viral resistance, abiotic stress, drought, tuber quality

## Abstract

Potato (*Solanum tuberosum* L.) is one of the most important food crops in the world, ranking fourth after rice, maize, and wheat. Potatoes are exposed to biotic and abiotic environmental factors, which lead to economic losses and increase the possibility of food security threats in many countries. Traditional potato breeding faces several challenges, primarily due to its genetic complexity and the time-consuming nature of the process. Therefore, gene editing—CRISPR-Cas technology—allows for more precise and rapid changes to the potato genome, which can speed up the breeding process and lead to more effective varieties. In this review, we consider CRISPR-Cas technology as a potential tool for plant breeding strategies to ensure global food security. This review summarizes in detail current and potential technological breakthroughs that open new opportunities for the use of CRISPR-Cas technology for potato breeding, as well as for increasing resistance to abiotic and biotic stresses, and improving potato tuber quality. In addition, the review discusses the challenges and future perspectives of the CRISPR-Cas system in the prospects of the development of potato production and the regulation of gene-edited crops in different countries around the world.

## 1. Introduction

Potato (*Solanum tuberosum* L.) is the fourth most important food crop in the world in terms of adaptability, yield potential, and nutritional advantages [1,2,3]. Despite their importance, potatoes are affected by biotic and abiotic stresses, which suppress their growth, development, and yield. Varieties resistant to diseases and environmental stress have been created using traditional breeding methods [4], but these breeding processes are slow due to the length of the stages involved. Historically, traditional breeding methods used to improve potato traits have mostly focused on yield, processing, and storage qualities [5,6,7]. Additionally, conventional breeding methods have been used to enhance agronomic traits [8] and processing [9], and breeding combined with molecular approaches has been used to improve storage quality [10] and disease resistance [11]. Moreover, potato breeders have improved its resistance to fungal diseases, such as late blight, by crossing cultivated potato (*S. tuberosum* L.) with wild species, including *S. brevidens* and *S. bulbocastanum* [12,13].

Although conventional breeding has been successfully applied to target the improvement of traits with less intraspecific variability, progress is relatively slow. Due to the complex breeding process, the development of new potato varieties takes approximately 10 years and is limited by the need for phenotypic characterization in subsequent generations. In addition, marker-assisted selection (MAS) has been used for more efficient selection in plant breeding [2,14]. Additionally, the introgression of beneficial genetic variation into cultivated potatoes is challenging due to the high heterozygosity and tetraploid nature of the cultivated potato genome [15]. Other disadvantages include intraspecific incompatibility and inbreeding depression, which lead to failures in traditional breeding [5]. In summary, the conventional breeding and genetic analysis of cultivated potatoes are associated with considerable difficulties associated with their tetrasomic inheritance, high heterozygosity, and self-incompatibility. In this context, new breeding technologies are required to improve potato crops and provide a platform for precise and efficient plant gene editing (GE).

GE enables the precise and efficient modification of genomic loci to create elite crop varieties with desired traits [16]. Recently used GE methods include zinc finger nucleases (ZFNs), transcription activator-like effector nucleases (TALENs), and clustered regularly interspaced short palindromic repeats (CRISPR)-associated proteins (CRISPR-Cas) [17,18,19]. Among GE technologies, CRISPR-Cas has the advantages of ease of operation, high efficiency, and low cost, demonstrating its potential in breeding resistant potato varieties [20].

CRISPR-based tools provide a diverse toolkit for GE, including DNA-targeted knockout, CRISPR-mediated transcriptional activation (CRISPRa) [21], CRISPR interference (CRISPRi) [22,23], base editing (BE) [24], prime editing [25], gene knock-in [26], and RNA targeting. A few of these tools have been successfully used for GE in potatoes, as will be described in this article. In its most common form, CRISPR-Cas9 technology enables gene knockout through targeted DNA editing. This involves using a guide RNA to direct the Cas9 enzyme to a specific DNA sequence, where it creates a double-strand break (DSB). The cell’s repair mechanisms then introduce mutations, often insertions or deletions (indels), at the break site, disrupting the gene’s function. The two major pathways for repairing DNA DSBs are homologous recombination (HR) and non-homologous end joining (NHEJ). The basic mechanism of GE is to create DSBs at a specific point using a template in the genome and utilize the host NHEJ mechanism to create indel mutations and subsequent gene knockout [27]. Most studies using CRISPR-Cas9 technology are based on the knockout of specific genes to produce a particular beneficial phenotype [28,29,30]. Meanwhile, BE is a GE method that allows precise, targeted changes to DNA sequences, modifying one base into another without creating DSBs [24]. Finally, RNA-targeting CRISPR–Cas systems are designed to target and cleave specific RNA sequences, primarily using Cas13 enzymes and CRISPR-guided RNA (crRNA). This method enables the modification or suppression of genes, as well as enhanced resistance to viral infections, by acting at the RNA level [31].

GE technology enables multiplex genetic engineering, significantly advancing crop research and designer crop development [32]. In autopolyploid crops, such as potatoes, CRISPR-Cas-based genetic engineering technology has been used to manipulate various important traits, including disease resistance by targeting genes involved in viral replication, susceptibility factors, and by introducing resistance genes [33].

Genetic engineering of plants using Cas9 can be achieved by various delivery methods, among which the most popular are *Agrobacterium*-mediated delivery, viral vector-mediated delivery, particle bombardment, and polyethylene glycol-mediated (PEG) delivery of ribonucleoproteins (RNPs). *Agrobacterium*-mediated GE shows promising efficiency and can be applied to most plant crops; however, transgene stability is achieved through fragment integration into the genome. Therefore, unintended background expression of the CRISPR-Cas cassette is expected to occur after GE, which may cause undesirable off-target effects compared to other delivery methods, such as viral vectors. Other DNA-free methods that allow transient expression can be used, albeit with other downsides [34]. One example of DNA-free delivery is CRISPR-Cas9 RNP delivery, which typically utilizes isolated protoplasts of plants grown in vitro, incubated in various concentrations of PEG 4000, and saturated with plasmid DNA or RNP containing gRNAs [35]. The RNP delivery method requires cell suspension and protoplast isolation, followed by whole-plant regeneration from the transfected protoplasts, to produce genetically modified plants. The particle bombardment method uses gold or silver particles as a carrier for vectors that contain the CRISPR-Cas9 construct. This method uses a gene gun to shoot these particles into plant tissue, delivering the constructs under high pressure. Particle bombardment has no biological limitations and can transform a wide range of tissue types. DNA-free methods of delivery have become widespread and have fewer regulatory and ethical concerns due to their DNA-free editing capabilities. Transgene-free potatoes have been obtained by knocking out various genes, including *granule-bound starch synthase* (*StGBSS*) [36] and *ALS* [37]. However, the effectiveness of GE depends on the success of regenerating whole plants from the targeted protoplasts, which is a time- and resource-consuming process [38]. These transgene-free methods are generally considered less effective compared to *Agrobacterium*-mediated transformation. For example, a study on maize showed an efficiency range of 0.85 to 5.85% [39], and another study on potato showed an efficiency of 2–3% [35]. 

Recently, viral vectors are expected to efficiently introduce CRISPR reagents into germ cells, allowing the editing of genes that do not contain heritable DNA. Due to their high replication rate and ability to effectively penetrate plant tissues, viruses are a convenient platform for the expression of guide RNAs (gRNAs) and Cas proteins. The use of RNA interference (RNAi) suppressors to enhance the efficiency of gene delivery and expression is currently being investigated. For example, modified versions of the P19 suppressor protein [39] can regulate both the expression level and the stability of gRNAs in plant cells. Combining viral vectors with such suppressors results in a more controlled and highly efficient delivery system, which is especially important for multiple or tissue-specific GE. Traditionally, genetic modification is achieved through the stable expression of CRISPR gene cassettes and the regeneration of transgenic lines. Delivery of autonomously replicating viral vectors into plants via *Agrobacterium* provides an alternative option for genetic editing of plant genes. In contrast, RNA viruses do not integrate into the plant genome; however, their limited transport capacity precludes the use of Cas9 [40]. One experimental work demonstrated *Agrobacterium*-mediated transfer of *geminivirus* vectors into potato leaf explants. *Geminiviruses* are a family of plant viruses with a single-stranded, circular DNA of approximately 2.5 to 3.0 kb. In the study by Acha et al., viral replicons and components containing Cas9 and gRNAs were integrated into the virus and replicated in the plant nucleus, resulting in effective genome edits. This transduction via *geminivirus* or other viral vectors can be potentially refined for stable integration and expression of CRISPR-Cas edits with no foreign DNA inserts [41]. Overall, the use of virus-based vectors for GE offers a promising method for crop breeding and functional genomic research.

The CRISPR-Cas tool has been successfully used previously to enhance potatoes’ tolerance to abiotic stresses, such as drought and salinity, and improve tuber quality by manipulating their amylose and amylopectin content and by reducing steroidal glycoalkaloids and enzymatic browning. GE, particularly CRISPR-Cas technology, enables more precise and rapid changes to the potato genome, thereby accelerating the breeding process and developing more effective varieties. In this review, we consider CRISPR-Cas technology as a potential tool for plant breeding strategies to ensure global food security. This review summarizes in detail the current and potential technological breakthroughs that open new opportunities for the use of CRISPR-Cas technology for potato breeding, as well as for increasing resistance to abiotic and biotic stresses, and improving potato tuber quality. In addition, this review discusses the challenges and future perspectives of the CRISPR-Cas system in the prospects of the development of potato production and the regulation of gene-edited crops in different countries around the world.

## 2. Using CRISPR-Cas Gene Editing Technology to Improve Potato Breeding Process

The presence of four copies (alleles) of genes in the tetraploid (2n = 4x = 48) genome of potato (*S. tuberosum*) makes it difficult for researchers/breeders to precisely edit the genome using traditional breeding tools [42]. Therefore, there are several approaches for enhancing the efficiency of potato breeding. One approach involves overcoming incompatibility and creating homozygous lines, which enable the rapid selection of lines with the desired, economically valuable traits. Following this, crossing the resulting homozygous lines can achieve hybrid vigor, which can be consolidated through vegetative propagation via tubers. A different approach involves improving various breeding traits in cell and tissue culture. Genetic and biotechnological methods are used to obtain the desired qualities in plants in vitro. These techniques enable the breeding process to be accelerated, thereby resulting in varieties and lines with the desired characteristics being obtained within a shorter timeframe.

**Figure 1:**
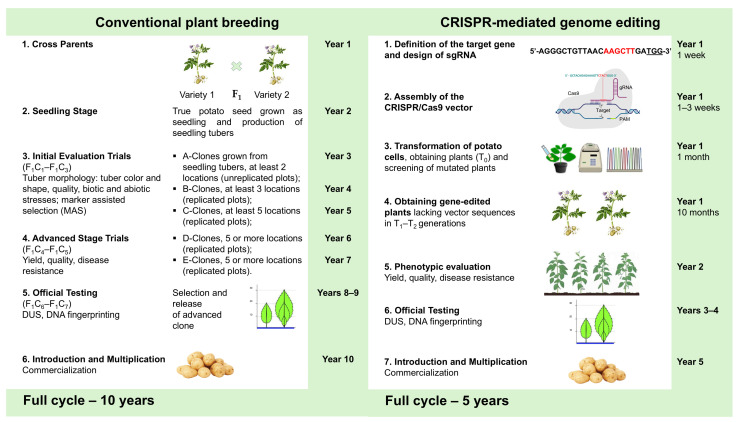
Conventional plant breeding (**left panel**) and CRISPR-Cas editing technology (**right panel**) for the potato breeding process. Conventional plant breeding (**left panel**): (1) Year 1—cross parents: selection of parental varieties, hybridization, berry formation, and true potato seed (TPS) extraction; (2) Year 2—seedling stage: the true potato seed grown as a seedling and the production of seedling tubers; (3) initial evaluation trails: Year 3—A-Clones (F_1_C_1_), grown from seedling tubers, grown at 2 locations on unreplicated plots; Year 4—B-Clones (F_1_C_2_), grown on at least 3 locations (replicated plots); Year 5—C-Clones (F_1_C_3_), grown on at least 5 locations (replicated plots); from Year 3 to Year 5 (F_1_C_1_-F_1_C_3_)—tuber morphology evaluation: tuber color and shape, quality, and resistance to biotic and abiotic stresses by Marker Assisted Selection (MAS); (4) advanced stage trails: Year 6—D-Clones (F_1_C_4_), grown on 5 or more locations (replicated plots); Year 7—E-Clones (F_1_C_5_), grown on 5 or more locations (replicated plots); from Year 5 and 6 (F_1_C_4_–F_1_C_5_)—phenotypic evaluation, yield, quality, and disease resistance; (5) official testing: Year 8–9 (F_1_C_6_–F_1_C_7_)—selection, distinctness, uniformity, and stability (DUS) testing, DNA fingerprinting, and the release of an advanced potato clone; and (6) introduction and multiplication: Year 10—commercialization of an advanced potato clone. Conventional potato breeding using MAS may take 10 years to develop a potato variety. CRISPR-mediated GE (**right panel**): (1) definition of the target gene and design of sgRNA (Year 1, 1 week); (2) assembly of the CRISPR/Cas9 vector (Year 1, 1–3 weeks); (3) transformation of potato cells and the obtaining and screening of mutated plants (T_0_) (Year 1, 1 month); (4) obtaining gene-edited plants lacking vector sequences in T_1_–T_2_ generations (Year 1, 10 months), and screening of CRISPR-Cas mutated plants using Sanger sequencing, CAPS analysis, or T7 assays, etc; (5) phenotypic evaluation—after confirming gene edits in the plants, phenotypic evaluation stage proceeds as in conventional breeding to evaluate yield, quality, and disease resistance (Year 2); (6) official testing: Year 3–4—selection, DUS testing, DNA fingerprinting, and the release of the edited potato variety; and (7) introduction and multiplication: Year 5—commercialization of the edited potato variety. The development of a potato variety may take 5 years; however, crop improvement using CRISPR-Cas technology is limited due to its cost, low efficiency, and regulatory issues.

Self-incompatibility is controlled by genetics through the highly polymorphic S-locus, which includes closely linked genes known as pistil-expressed S-RNase (S-locus RNase) and pollen-expressed F-box protein (*SLF* or *S*-locus F-box). Pollen tube growth is one of the significant indicators of self-incompatibility, and multiallelic RNase in the pistil blocks is incompatible with pollen tube growth [16]. In previous studies, scientists created a self-compatible diploid potato by knocking out the self-incompatibility gene S-RNase using CRISPR-Cas9 (Table 1, Figure 1) [43,44]. Later, self-compatibility was achieved in nine S-RNase KO T0 lines that contained biallelic and homozygous deletions/insertions in both genotypes, transmitting self-compatibility to T_1_ progeny [45]. The *S*-locus inhibitor (*Sli*) gene was introduced using the CRISPR-Cas9 system. A 533 bp insertion fragment was found in the promoter region of the *Sli* gene, allowing it to be expressed in pollen. In future breeding efforts, this region could be introduced into the *Sli* gene promoter using targeted insertion [46].

Zong et al. used PBE and A3A-PBE to generate six base-editor constructs (T1 to T6) targeting *StGBSS* via PEG-mediated protoplast transfection. In their results, two independent heterozygous mutant potato plants were recovered at the StGBSS-T6 target site from 31 regenerated plants. None of the transgenic potato plants contained indels or undesired edits at the target sites. To the knowledge of the authors, this was the first report of base-editing via cytidine deamination in potato, which should pave the way for the widespread use of *A3A-PBE* in dicotyledonous plants [47].

The potato *acetolactate synthase 1* (*StALS1*) gene was edited by Butler et al. to test the induction of targeted mutations in the callus and stable events of diploid and tetraploid potatoes. Targeted mutations ranged from 3% to 60% per transformation and from 0% to 29% above the expected threshold, based on the number of *ALS* alleles [20]. To overcome the barriers associated with gene targeting, a *geminivirus* replicon (GVR) was used to deliver SSNs targeting the potato *acetolactate synthase 1* (*StALS1*) gene and repair templates designed to incorporate herbicide-inhibitory point mutations into the *StALS1* locus. GVR-modified transformations contained point mutations that could maintain a reduced herbicide susceptibility phenotype. These results demonstrate the use of *geminiviruses* to deliver genome-editing reagents into plant species and a new approach to gene targeting in vegetatively propagated species [48]. Das Dangol et al. showed that targeting the invertase inhibitor (*StINVINH1*) affected the rate of callus induction and regeneration, was effective under heat stress conditions, and reduced its gene expression levels [49].

Hairy root transformation using *Agrobacterium rhizogenes* provides an accelerated approach to generating transgenic material [50]. Recently, hairy root transformation has been established as an effective tool for studying gene function using CRISPR-Cas9 GE in a wide range of plant species [52]. In a study by Butler et al., *A. rhizogenes* strains were tested on a wild diploid relative of potato, *Solanum chacoense*, which is recalcitrant to *Agrobacterium tumefaciens* infection. CRISPR-Cas9 reagents targeting the *potato phytoendesaturase gene* (*StPDS*) were expressed in hairy root clones and regenerated. A total of 64–98% of transgenic hairy root clones were found to carry the target mutations, while only 14–30% of the mutations were chimeric [50]. In the study by Eggers et al., mutations were maintained in the regenerated lines as stable mutations at an average frequency of 38% and were capable of being transmitted to the progeny through the germline. This new approach expands the number of genotypes amenable to *Agrobacterium*-mediated transformation while reducing chimerism in primary events and accelerating the generation of edited materials [46].

The *Erecta* family of genes (*ERfs*) is involved in the regulation of epidermal development and plant growth. The *StER* gene also enhances plant growth. To miniaturize plant architecture, Xin et al. developed two “mini-Jan” lines with compact and dwarf plant growth using CRISPR/Cas9-based mutagenesis targeting the growth-related *dwarf* and *Erecta* genes. One mini-Jan mutant, mini-JanE, is fully fertile and can enable more throughput studies. Jan and mini-Jan offer a robust model system that can be used for GE and functional genomics studies in potato [51].

Works dedicated to self-incompatibility have uncovered the potential for potato breeding both at the tetraploid and diploid levels, as they expedite the creation of homozygous lines and thus facilitate the breeding process. Therefore, the use of CRISPR-Cas technology will significantly increase the efficiency of obtaining valuable forms/lines for breeding, and the frequency of such work will increase, especially concerning potato breeding in the future. The authors of this review believe that research on cell and tissue culture will expand in the future. Because potatoes are very responsive to regeneration in cell and tissue culture, the combination of CRISPR-Cas technology and tissue/cell culture has the potential to increase the efficiency of the potato-breeding process.

## 3. Using CRISPR-Cas Gene Editing Technology to Increase Disease Resistance in Potato

Potato plants are exposed to various biotic stresses [53], including late blight [54], viral diseases [55], potato cyst nematode [56], bacterial wilt [57], soil-borne diseases [58], and tuber diseases [59].

**Figure 2:**
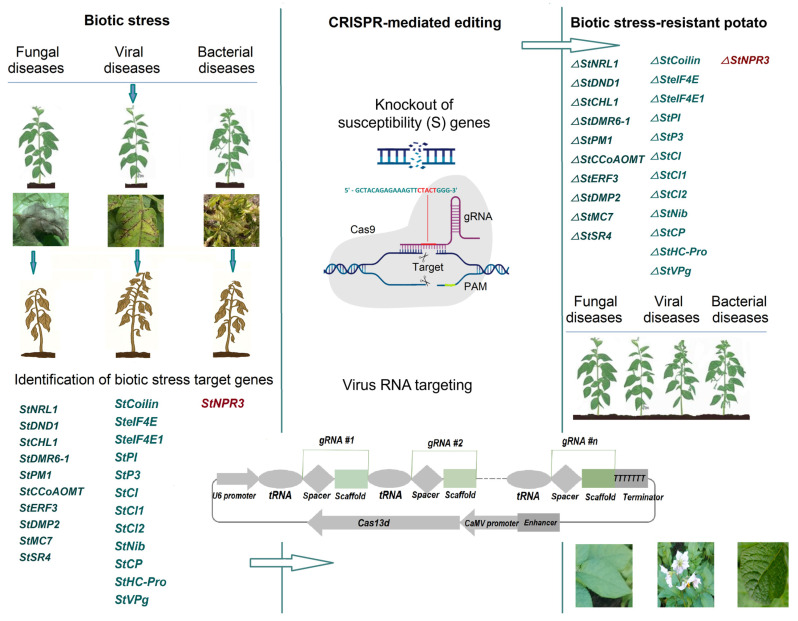
Using CRISPR-Cas technology to improve potato resistance to biotic stresses. Biotic stress includes fungal, viral, and bacterial diseases. In wild type potato (**left panel**) *StNRL1*, *StDND1*, *StCHL1*, *StDMR6-1*, *StPM1*, *StCCoAOMT*, *StERF3*, *StDMP2*, *StMC7*, and *StSR4* genes negatively regulated late blight resistance, while StDMR6-1 negatively regulated late blight, early blight, and common scab; *StCoilin*, *SteIF4E*, *SteIF4E1*, *StP3*, *StCI*, *StNib*, *StCP*, *StPI*, *StHC-Pro*, *StCl1*, *StCl2*, and *StVPg* genes negatively regulated resistance to potato virus Y, while CP multiplex virus resistance PVY, PVS, PVX, or PLRV; *NPR3* gene negatively regulated resistance to *Clso.* In CRISPR-Cas edited plants (**right panel**) knockout of susceptibility (S) genes (Δ*StNRL1*, Δ*StDND1*, Δ*StCHL1*, Δ*StDMR6-1*, Δ*StPM1*, Δ*StCCoAOMT*, Δ*StERF3*, Δ*StDMP2*, Δ*StMC7*, Δ*StSR4*, Δ*StCoilin*, Δ*SteIF4E*, Δ*SteIF4E1*, Δ*StNPR3*) and virus RNA targeting (Δ*StP3*, Δ*StCI*, Δ*StNib*, Δ*StCP*, Δ*StPI*, Δ*StHC-Pro*, Δ*StCl1*, Δ*StCl2*, Δ*StVPg*) improved resistance to biotic stresses. The loss of function genes Δ*StNRL1*, Δ*StDND1*, Δ*StCHL1*, Δ*StDMR6-1*, Δ*StPM1*, Δ*StCCoAOMT*, Δ*StERF3*, Δ*StDMP2*, Δ*StMC7*, and Δ*StSR4* improve resistance to *P. infestans.* The loss-of-function gene Δ*StNPR3* improves resistance to *Clso*. The loss-of-function genes Δ*StCoilin*, Δ*SteIF4E*, Δ*SteIF4E1*, Δ*StP3*, Δ*StCI*, Δ*StNib*, Δ*StCP*, Δ*StPI*, Δ*StHC-Pro*, Δ*StCl1*, Δ*StCl2*, and Δ*StVPg* improve resistance to potato virus Y. The Δ*StCP* gene improves multiplex virus resistance to PVY, PVS, PVX, and/or PLRV.

Late blight is a serious disease that affects the growth rate and quality of potato tubers and is caused by the *Phytophtora infestans* fungus. Its impact can reduce potato yields significantly by up to 80% if proper control measures are not taken. This disease can also cause complete tuber rot, posing a significant threat to food security, particularly in low-income and developing countries [60]. The use of chemical fungicides is the most common control strategy for late blight, but the emergence of new fungicide-resistant isolates poses a challenge to plant breeders. The most effective way to control *P. infestans* is to develop resistant potato varieties [54]. However, the development of resistant varieties is a lengthy process, so using GE methods is the fastest and most effective way to create varieties that are resistant to late blight. Plant breeders aim to improve disease-resistant crops by either pyramiding resistance (*R*) genes or by knocking out susceptibility (*S*) genes. With the help of modern GE approaches, knockout of *S* genes is an effective way to reduce plant susceptibility to diseases [54,61,62]. The CRISPR-Cas9 system was used as a tool for knocking out the *StNRL1 S* gene in potato. As a result of this work, it was demonstrated that a mutant line with a four-allelic knockdown of the *StNRL1* gene, resulting in a decrease in *StNRL1* expression level of approximately 90%, exhibited increased resistance to *P. infestans*. Surprisingly, the mutant lines were susceptible to *Alternaria alternata*, suggesting that *StNRL1* may play a role as a resistance gene; therefore, downregulation of *StNRL1* enhances resistance to *P. infestans* [54]. One typical *S* gene is *MLO* (*Mildew Locus O*), which was initially characterized in spring barley in the 1940s and later used in European plant breeding programs in the 1970s [63]. Because they confer nonspecific, long-lasting resistance under field conditions, *MLOs* have been used in a wide range of plant crops [64,65]. The first group of *MLOs* includes genes required for host recognition by the pathogen. The second group includes genes that support pathogen requirements, such as the SWEET sugar transporters. The third group of *MLOs* includes genes that control plant defense responses. Candidate *S* genes include *downy mildew resistance 6* (*DMR6*) and *defense no death 1* (*DND1*). Many *S* genes encode negative regulators of plant defense responses, such as DMR6. In a more recent study using the CRISPR-Cas9 system that co-expressed two guide RNAs, tetra-allelic deletion mutants were generated, and plant resistance to late blight was investigated. Functional knockouts of the *StDND1, StCHL1,* and *StDMR6-1* genes resulted in enhanced resistance to late blight. However, plants with mutated *StDND1* demonstrated pleiotropic effects, whereas plants with mutated *StDMR6-1* and *StCHL1* did not show any growth phenotypes, making them promising candidates for further agricultural research [62]. Recently, Karlsson et al. conducted a field evaluation of the *StDMR6-1* knockout mutants exposed to a *P. infestans* population over four years, finding an enhanced resistance to late blight without any compromise in terms of yield or tuber quality loss. In addition, studies of potato tubers from field trials have shown increased resistance to common scab, and mutant lines have demonstrated increased resistance to the pathogen *Alternaria solani* under controlled conditions. These studies have shown that targeting *StDMR6-1* alone does not solve the potato late blight problem; other integrated pest management measures need to be used [66]. A novel susceptibility factor in potato, plasma membrane protein 1 (*StPM1*), is encoded by the *ABA-induced wheat plasma membrane polypeptide-19* (*AWPM-19*)-like family and is involved in the defense response to *P. infestans.* It was demonstrated that knocking out the *StPM1* gene using CRISPR-Cas9 enhanced potato resistance to late blight without affecting potato growth and development (Table 2, Figure 2). These findings suggest that the modification of *S* genes in crop plants may open new avenues for enhancing plant disease resistance [67]. The successful correction of the *StCCoAOMT* gene knockout mutation in Russet Burbank using CRISPR-Cas9 resulted in significant reductions in disease severity and pathogen biomass in pathogen-inoculated knockout plants compared to unedited plants [68]. Plant–pathogen interactions lead to the molecular modulation of stress signals through signaling elements that activate various stress-responsive genes. The products of these genes may play a role in the production of regulatory phytohormones (ethylene, salicylic acid, or abscisic acid), which can then induce the resulting plant response. A key plant defense hormone is ethylene, which plays a role in growth and development. Specifically, it modulates the expression of defense genes through transcription factors known as *ethylene response factors* (*ERFs*). In their study, Razzaq et al. turned off the function of *StERF3* in potatoes using GE. Silencing the potato *ERF3* gene resulted in increased resistance to *P. infestans*, thus identifying this approach as the most effective way to strategically control late blight in potato plants [69]. The *domain of unknown function 679 membrane protein* (*DMP*) family positively regulates resistance to late blight. Specifically, a member of this family in potatoes, *StDMP2*, is localized to the endoplasmic reticulum (ER) and is shown to positively regulate resistance to *P. infestans*. In addition, *StDMP2* affects the accumulation of specific *pattern recognition receptors* (*StPRRs)* and *non-race-specific disease resistance 1* (*StNDR1*). Research results show that *StDMP2* positively regulates plant resistance [70]. Metacaspases (MCs) are involved in programmed cell death (PCD) in plants during development, as well as during resistance to abiotic and biotic stresses. Knockdown of the *StMC7* gene under induced infection with *A. solani* and *P. infestans* decreased plant susceptibility in mutants compared to the wild type [71]. *Signal Response 1* (*SR1*), also known as calmodulin (CaM)-binding transcription activator 3 (CAMTA3), is a calmodulin-binding transcription factor that plays a critical role in plant stress responses and ethylene signaling. Knockout of the *StSR4* gene induces salicylic acid synthesis, thereby increasing plant resistance to late blight [72].

Potato zebra chip disease is a potato disease that causes significant economic losses worldwide. The etiologic agent of potato zebra spot is *Candidatus Liberibacter solanacearum* (Clso), and it is transmitted to potato plants by the psyllid *Bactericera cockerelli* [80]. In plants, *non-expressors of pathogenesis-related* (*NPR*) proteins function as receptors for salicylic acid. Ramasamy et al. proposed a working model in which, in potatoes, the knockdown or complete deletion of *StNPR3* activates salicylic acid signaling and increases resistance to *C.L. solanacearum*. Potato *NPR3* plays a vital role in regulating salicylic acid–jasmonic acid homeostasis and represents a strategy for achieving disease resistance by disrupting its function using GE technology [73].

Potato viral diseases significantly reduce crop yields worldwide. There are approximately 40 types of viral diseases that affect potatoes. Due to their global importance, controlling the spread of these diseases is one of the most pressing problems the world faces [46]. The *coilin* gene in potato plays a key role in PVY infection. Editing at least one allele of the *coilin* gene significantly increased the resistance of edited lines to potato virus Y infection and their tolerance to salt and osmotic stress [74]. One of the primary targets is a group of genes belonging to the family of *eukaryotic translation initiation factors* (*eIFs*). The potato *eIF4E* gene family comprises *SteIF4E1* and its paralogs *SteIF4E2*, *SteIF(iso)4E*, and *StnCBP*. The homozygous conserved region of *SteIF4E* in the potato variety Kruda was mutated using CRISPR-Cas9. The loss of function of the *SteIF4E* gene resulted in increased resistance to PVY in potato [75]. The expression of a modified eukaryotic translation initiation factor (*eIF4E*) also provided resistance to PVY [81]. Similarly, in a study by Lucioli et al., the tetraploid potato variety Desirée, which harbors a dominant *Ny* gene conferring resistance to the O strain of potato virus Y, but not NTN, was used to evaluate the possibility of broadening the potato’s PVY resistance spectrum via the CRISPR-Cas9-mediated knockout of the susceptibility gene *SteIF4E1*. The knockout of *SteIF4E1* can broaden the resistance spectrum of the elite variety “Desirée” conferred by the *Ny* gene of PVYO, and knockout of *SteIF4E1* does not alter the steady-state mRNA levels of other potato *eIF4E* gene family members [76]. Editing *SteIF4E1* can be used to broaden the spectrum of PVY resistance in potatoes by pyramiding the recessive resistance mediated by *SteIF4E* [76,82]. Regarding genetically engineered resistance based on RNA silencing, the basal antiviral plant defense system, together with CRISPR-Cas13a, can provide protection against PVY in potatoes [83]. In addition to RNAi [84], recent advances in CRISPR-Cas system-mediated DNA or RNA editing/interference in plants have made them key components of plant defense [85]. CRISPR-Cas13 was used to confer broad-spectrum resistance in transgenic potato plants. Zhan et al. engineered high-level PVY resistance in potato plants by engineering sgRNA targeting *StP3*, *StNib*, or *StCP* coding sequences in the genomic region of potato virus Y; this CRISPR-Cas13a system also showed efficacy in hindering and inhibiting PVY infection. Importantly, CRISPR-Cas13a technology enables the targeting of viral genomes and can, therefore, be used to target multiple PVY strains [77]. Cas13d has also been used to detect multiplex RNA viruses in potatoes, making it an effective tool for enhancing agricultural productivity and sustainability. Zhan et al. developed a Cas13d/PTG system to engineer broad-spectrum resistance to multiple RNA viruses in potatoes. The gRNAs targeting the PVY genome were organized in the Cas13 RNA cassette as *StPI*, *StHC-Pro*, *StP3*, *StCl1*, *StCl2*, and *StVPg*. It has been demonstrated that the CRISPR-Cas13a system can be engineered to confer broad-spectrum resistance to multiple PVY strains in potato plants [78]. The Cas13d/PTG system enables programmable RNA virus interference to target either a single virus or two/three mixed RNA viruses simultaneously, thereby expanding the applicability of the CRISPR system to crop protection against multiple RNA viruses [79]. The targeted knockout of the *StCoilin* gene, the mutation of the *SteIF4E* gene families, and the expression of LshCas13a/sgRNAs and Cas13d/PTG may be effective in conferring broad resistance to multiple viral diseases in potatoes.

The development of herbicide-tolerant varieties has become a significant area of focus in potato breeding. However, the genetic improvement of herbicide tolerance in potatoes is challenging due to the limited number of herbicide tolerance genes available for use in breeding. The bacterial enzyme 5-enolpyruvylshikimate-3-phosphate synthase (EPSPS) has a slight modification that prevents glyphosate binding, thereby allowing the plant to continue producing amino acids even in the presence of glyphosate. Bakhsh et al. transferred the *EPSPS* gene from an *Agrobacterium* strain into potatoes, resulting in transgenic plants derived from these varieties that exhibited enhanced glyphosate tolerance in both the T0 generation and the first tuber generation. These transgenic potato lines then provided a source of germplasm for an efficient potato breeding program [86].

Potatoes are exposed to various biotic stressors that affect yield and quality, among which potato diseases are the most prominent. The use of insecticides increases the population of resistant vectors and has detrimental effects on the environment. GE via CRISPR-Cas is a crucial research tool and a means for the genetic improvement of potato varieties and lines, as it enables the precise introduction of targeted genetic changes in the desired resistance and susceptibility genes. Several studies have been conducted to confer potato resistance to fungal (late blight, early blight, and common scab), viral (PVY, PVS, PVX, or PLRV), and bacterial diseases (zebra chip). Moreover, the emergence of different CRISPR-Cas versions enhances the effectiveness of GE in potato through the gain and/or loss of gene functions. The availability of the complete genomic sequence of potato allows scientists of the post-genomic era to design its genome specifically to improve traits such as disease resistance and increased productivity, thereby enhancing the agronomic parameters of the tuber. CRISPR-Cas-based technology is making progress toward enhancing disease and pest resistance and has enormous potential to address critical agricultural and environmental issues worldwide, ensuring sustainable global food security.

## 4. Using CRISPR-Cas Gene Editing Technology to Increase Abiotic Tolerance in Potato

**Figure 3:**
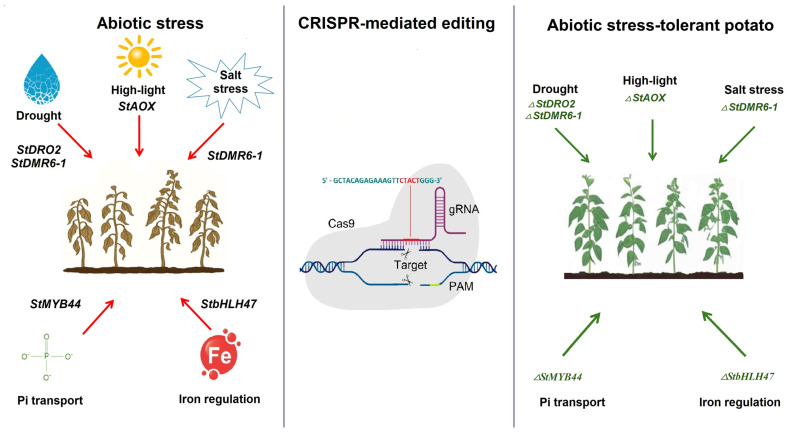
Using CRISPR-Cas technology to improve potato tolerance to abiotic stresses. Abiotic stresses include drought, salinity, high-light, Pi transportation, and iron regulation. In wild type potato (**left panel**), genes involved in negative regulation include the following: drought (*StDMR6-1*, *StDRO2*); high-light (*StAOX*); salt stress (*StDMR6-1*); Pi transportation (*StMYB44*), and iron regulation (*StbHLH47*). In CRISPR-Cas edited plants (**right panel**), knockout of target genes for drought (Δ*StDMR6-1*, Δ*StDRO2*), high-light (Δ*StAOX*), salt stress (Δ*StDMR6-1*), Pi transportation (Δ*StMYB44*), and iron regulation (Δ*StbHLH47*) improved resistance to abiotic stresses. The loss of function of the following genes: Δ*StDMR6-1*—improved drought and salt tolerance; Δ*StDRO2* and Δ*StAOX*—high-light tolerance; Δ*StMYBb44*—improved Pi transportation; and Δ*StbHLH47*—iron regulation. Nonsense mutation of Δ*StDRO2* improved drought tolerance.

The most important abiotic stresses affecting potato productivity worldwide are drought, salinity, suboptimal temperatures, and high-light levels. Drought significantly increases the costs of agricultural production. Research on plant drought tolerance is becoming increasingly crucial for minimizing the impact of global climate change on agriculture, as most crops are grown in water-stressed regions of the world [53,87,88,89]. The broad resistance of StDMR6-1 mutants to *P. infestans* and *A. solani*, as described above, extends to improved resistance to several abiotic stress conditions. In the experiments of Karlsson et al., under control conditions, when simulating drought and salinity, the *StDMR6-1* mutant plants were less affected than the control plants [66]. Gene editing *StDMR6-1* thus shows promise as a valuable tool against disease and abiotic stresses, such as drought and salinity (Table 3, Figure 3). The *transcription factor* (*StbHLH47*) is involved in the regulation of drought tolerance and iron homeostasis. Wang et al. identified the *StbHLH47* gene and showed that *StbHLH47* negatively regulates drought stress tolerance in potato. These results suggest that the *StbHLH47* gene can be used to enhance plant tolerance to abiotic stresses and iron homeostasis [90]. However, studies by Chaugan et al. showed that editing the *StbHLH47* gene negatively regulates the expression of genes related to iron homeostasis. Reduced FCR activity, phenotype changes, and increased iron content in potato plants were found in *StbHLH47*-edited lines [91]. The *DRO1* gene controls root growth and enhances plant adaptation to drought. Studies have shown that *StDRO2* can affect auxin transport, and that the controlling effect of *StDRO2* on RSA can be regulated by auxin and microRNA. The results indicated that the *StDRO2* gene may play an important role in potato root formation [92].

Abiotic stresses also induce the activation of the cyanide-resistant mitochondrial respiratory pathway in potatoes. Plant alternative oxidase (AOX) is a functional protein involved in the process of cyanide-resistant respiration, and AOX expression can be activated by developmental changes and stresses, such as harsh light. High-light stress causes stomatal closure, decreased photosynthesis, the accumulation of reactive oxygen species (ROS), and accelerated respiration. AOX, as an active oxygen scavenger, is involved in plant responses to many stresses. The results obtained by Hua et al. show that, under severe light stress, potatoes with reduced *StAOX* content have activated antioxidant systems, which accelerate the malate–oxaloacetate shuttle pathway and photorespiration, thereby reducing the amount of excess reducing power in leaf cells. The presence of AOX is important for plants to maintain cellular homeostasis and ensure adequate photosynthesis under high-light conditions in the field [93].

Phosphorus is a vital macronutrient necessary for the growth of plants, yet it is frequently lacking in soil. To investigate the molecular mechanisms underlying the intricate responses of potato plants to phosphate (Pi) deficiency stress, an RNA-Seq methodology was employed to identify genes that react to Pi starvation in the roots of potato. The *S. tuberosum* transcription factor *MYB44* (*StMYB44*) gene was found to be downregulated by Pi starvation. The knockout of the *StMYB44* gene by the CRISPR-Cas9 system did not increase the transcription of *StPHO1*, and *StMYB44* was discovered to interact in the nucleus with AtWRKY6, a known transcription factor in *Arabidopsis* that directly regulates PHO1 expression, as well as with *StWRKY6*, suggesting that *StMYB44* might be a component of the regulatory complex that governs *StPHO1* transcription. Taken together, that study demonstrated that *StMYB44* negatively regulates Pi transport in potato by suppressing *StPHO1* expression [94].

With the growing global population, it is critical to use new technologies to develop crops that are tolerant to abiotic stresses. This article reviewed the advances made by scientists around the world in applying CRISPR-Cas technology for potato improvement. Several researchers have used gene knockout and editing in the potato genome to improve drought tolerance [66,92], high-light tolerance [93], and the regulation of phosphorus transport [94]. However, climate change exacerbates water stress and increases the frequency and intensity of extreme weather events, such as heat waves, cold waves, and drought, which makes it imperative to improve tolerance to these stressors. Thus, enhancing the resistance of potatoes to these stressors (drought, salinity, and low and high temperatures), in combination with optimal nutrition, may facilitate the acclimatization of potatoes in different agro-ecological zones, thereby preventing food shortages in less fertile regions or deficient agricultural land. Further research in the field of potato GE may enable new ways to increase yields, resistance to diseases, resistance to climate change, and improvement of the quality and nutritional value of this important crop.

## 5. Using CRISPR-Cas Gene Editing Technology for Potato Quality Improvement

CRISPR-Cas technology can be used to accelerate the breeding process and directly improve specific agronomic traits by editing individual genes without altering other parts of the genome. Important market traits include tuber appearance, shape, flesh color, skin texture, and skin color.

Potato tuberization is a key stage in crop development, determining the yield and quality of tubers. Increased expression of *StSN2* can induce potato tuberization and increase yield. Deletion of the *StSN2* gene leads to a delay in the formation of potato stolons and a decrease in yield of 20–30%. The results showed that *StSN2* enhances the ABA signaling pathway and thus enhances tuber formation [95]. The *cycling dof factor 1* (*StCDF1*) gene is a key gene in potato tuberization. Wan et al. used *StCDF1* and identified the photoperiod inducible region of the 288 bp core promoter. Delayed tuberization was noted as a result of two deletions in the core promoter [96]. *StSP6A* is also involved in the formation of potato tubers. The effect of the knockout of the key gene *StSP6A* on H_2_O_2_-induced tuber formation was studied to elucidate the role of H_2_O_2_ in the induction and formation of potato tubers. The results showed that *StSP6A* is a key element in H_2_O_2_-induced potato tuber formation [97].

**Figure 4:**
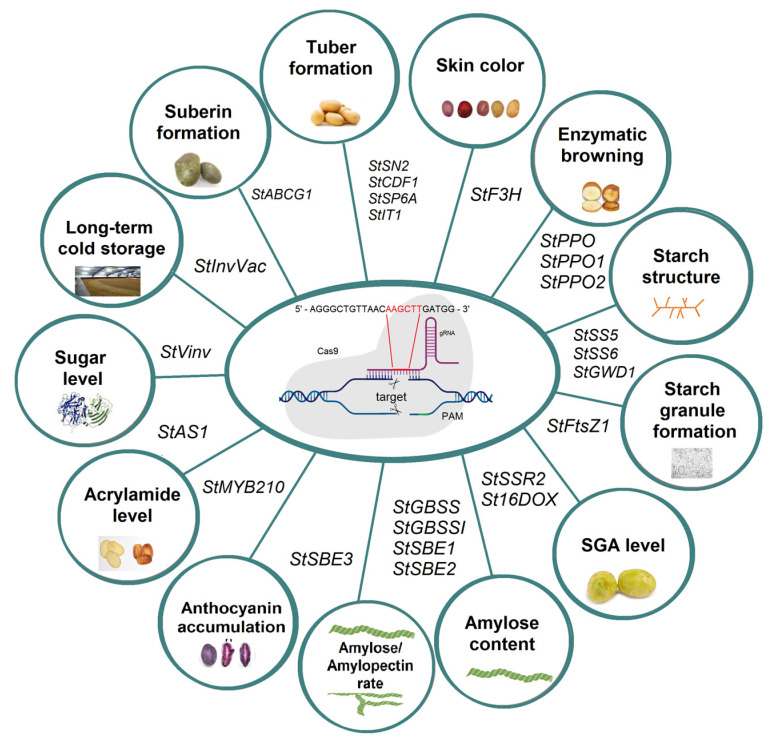
Using CRISPR-Cas gene editing technology for enhancement of potato qualities. In CRISPR-Cas edited plants, knockout of target genes, *StSN2 StCDF1*, *StSP6A* and *StIT1*, enhanced potato tuberization; *StF3H* gene was involved in tuber skin color change, from red to yellow; *StPPO*, *StPPO1*, and *StPPO2* genes reduced enzymatic browning; *StFtsZ1*, *StSS5*, and *StSS6* genes altered starch granule size; *StGWD1* gene improved starch granule; *StSSR2* and *St16DOX* genes reduced level of *SGA; StGBSS*, *StGBSSI*, *StSBE1*, *StSBE2*, and *StSBE3* genes regulated amylopectin/amylose ratio; *StMYB210* gene stopped anthocyanin synthesis; *StVinv* and *StAS1* genes reduced sugars and low acrylamide levels; *StVinv* gene reduced fructose and glucose concentrations after cold storage; *StInvVac* gene was responsible for long-term cold storage and bruising resistance; *StABCG1* gene decreased suberin production.

Wulff-Vester et al. changed the skin color of two potato varieties, Desirée and “Nansen, from red to yellow by using the CRISPR-Cas9 system to knockout flavanone 3-hydroxylase (F3H), a key enzyme in the anthocyanin pathway (Table 4, Figure 4). The results showed that yellow skin remained constant over three generations of tuber breeding [98]. The tuber flesh color is one of the most studied traits in potato. Flesh color can range from yellow to white and purple. Carotenoids and pigments, such as anthocyanins, determine the color of the flesh. Yellow-fleshed potatoes are known for higher levels of carotenoids, while purple-fleshed potatoes are rich in anthocyanins; white-fleshed potatoes are a source of essential nutrients like potassium, vitamin C, and fiber. The color of flesh can be altered using CRISPR-Cas technology by targeting genes involved in carotenoid and pigment production. Therefore, Tang et al. demonstrated that the *StIT1* gene, which is linked to carotenoid content, when mutated by CRISPR-Cas9, resulted in stolon branching, which laid the foundation for an increased yield [99].

Enzymatic browning during processing, storage, transportation, and shelf life can reduce the commercial value of potatoes and lead to economic losses [100]. CRISPR-Cas9 technology has been applied to develop potato varieties with reduced enzymatic browning by specifically editing one member of the *StPPO* gene family [101]. Editing the *StPPO2* gene resulted in reduced PPO activity in the tuber and a subsequent reduction in enzymatic browning. Mutations induced in four alleles of the *StPPO2* gene resulted in lines with an up to 69% reduction in tuber PPO activity and a 73% reduction in enzymatic browning compared to the control group [101,102]. Next, pGEF-StPPO1 and pGEF-StPPO2 were engineered to target the two most likely relevant PPO isoforms (*StPPO1* and *StPPO2*), which showed the efficacy of the corresponding pGEF-X vectors in editing these genes. The edited line for *StPPO1* allows functional evaluations to begin after tuber generation is achieved, revealing the power of this procedure in potato GE. The results support pGEF-U for GE in combination with regular regeneration protocols, and both targeted deletion and single-site editing encourage the further characterization of the set of already generated plants [41]. Moreover, the results obtained [41,101,103] do suggest that the specific editing of one member of the *StPPO* gene family can be applied to generate potato varieties with reduced enzymatic tuber browning. Jayakody et al. demonstrated CRISPR/Cas9 editing specificity by evaluating off-target editing outcomes using high-coverage whole-genome shotgun sequencing data. There was negligible CRISPR/Cas9 off-target activity compared to regular somatic background variation produced from tissue culture or spontaneous mutations [104]. There have been other studies validating these research results on other crops, such as cotton [105], grapevine [106], and rice [107].

**Figure 5:**
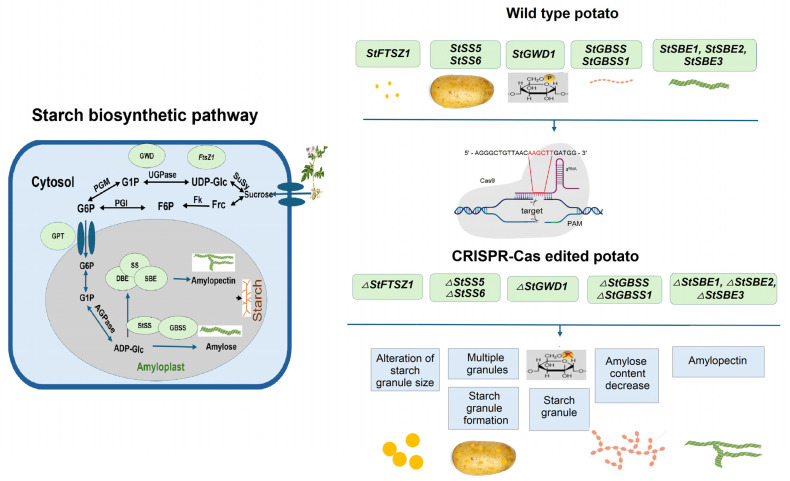
Using CRISPR-Cas gene editing technology to improve starch quality. The starch biosynthetic pathway is shown (**left panel**). In wild type potato (**right upper panel**), the *StFtsZ1* gene is involved in starch granule size; the *StSS5* and *StSS6* genes are involved in starch granule formation; the *StGWD1* gene catalyzes the phosphorylation of the glucose chain of starch; the *StGBSS* and *StGBSSI* genes are responsible for amylose content; the *StSBE1*, *StSBE2*, and *StSBE3* genes regulate amylose content. In CRISPR-Cas edited plants (**right lower panel)**, knockout of target genes included the following: Δ*StFtsZ1* gene altered starch granule size; Δ*StSS5* and Δ*StSS6* genes increased formation of multiple starch granules; Δ*StGWD1* reduced phosphorylation; Δ*StGBSS* and Δ*StGBSSI* decreased amylose content; and Δ*StSBE1*, Δ*StSBE2*, and Δ*StSBE3* genes increased amylopectin content.

Potato is an important source of starch for both food and industrial applications. Potato starch is composed of amylose and amylopectin. The amylose/amylopectin ratio affects both the dietary and industrial properties of starch [108]. Therefore, users of CRISPR-Cas technology have mainly focused on either producing amylose-free potatoes or on increasing the amylose content by editing the genes involved in starch biosynthesis. Starch granules are produced in the plastids of tubers, and plastid size is correlated with the size of the starch granules. The division of plastids is controlled by proteins, including the tubulin-like GTPase *FtsZ1* (Figure 5). Pfotenhauer et al. edited *StFtsZ1* to increase the size of starch granules in tubers. Several lines showing an up to 1.98-fold increase in starch granule size were generated, which were otherwise phenotypically indistinguishable from wild type plants. In addition, starch paste from one of the most promising lines showed a 2.07-fold increase in final viscosity [109]. Starch synthases can be further divided into soluble *starch synthase* (*SS*) and *granule-bound starch synthase* (*GBSS*). Within the starch biosynthesis pathway, the starch-bound proteins perform enzymatic functions. New isoforms of starch synthase, *StSS5* and *StSS6*, are key enzymes in the starch biosynthesis pathway. The inactivation of these enzymes can lead to changes in starch properties; these changes have potential in industrial applications [110]. Sevestre et al. used an SNP physical map of the widely used *Solanum tuberosum* L. cv. Desiree to, based on the position of the diverse indels, perform a knockout of the *StSS6* gene using CRISPR-Cas9. The transformed plants showed a very high probability of knockout mutation for each of the four *StSS6* alleles. Therefore, the inactivation of this enzyme may lead to changes in starch properties [111]. Similar work was performed by Hu et al., where knockdown of the *StSS5* gene in potato tubers increased the small granule content and improved starch yield [112]. α-*glucan*, *water dikinase 1* (*GWD1*) is an enzyme that catalyzes the phosphorylation of the glucose chain of starch. In the work of Ohnuma et al., the phosphorus content was significantly reduced in mutant plants, and the amylose content was higher compared to the wild type [113].

Reducing the content of steroidal glycoalkaloids (SGAs), such as α-solanine and α-chaconine, in tubers is a key requirement for breeding superior potatoes, as high contents of these compounds can impart a bitter taste and have potentially adverse effects on humans. SGAs, which are present in most potato tissues, are toxic to humans, and high SGA contents can also damage potato tuber quality. Sterol side chain reductase 2 (SSR2) is a key enzyme for SGA biosynthesis in potato. To reduce the SGA concentration in potatoes, the *StSSR2* gene was edited using the CRISPR-Cas9 system. The SGA content was significantly reduced in the edited potato lines. The knockout of *StSSR2* using the CRISPR-Cas9 system was found to reduce SGA levels in leaves and tubers effectively and to reduce the relative transcript levels of genes involved in steroidal glycoalkaloid biosynthetic pathways [30]. The St16DOX enzyme is a 2-oxoglutarate-dependent dioxygenase that catalyzes the 16α-hydroxylation of (22S)-22,26-dihydroxycholesterol in the later stages of SGA biosynthesis. Nakayasu et al. generated two SGA-null potato hairy root lines with *St16DOX* gene knockout via transformation, using a novel CRISPR-Cas9 vector pMgP237-2A-GFP designed to provide multiplexed gRNA expression based on the pre-tRNA processing system. The two lines were found to have multiple mutations at different sites and a deletion of the *St16DOX* chromosomal fragment [114]. Potato varieties with a higher amylopectin content, created by knocking down the amylose-producing gene *StGBSSI*, have multiple applications within the food industry. Abeuova et al. demonstrated the successful RNA-directed mutagenesis of the potato *GBSS* gene via CRISPR-Cas9, resulting in an amylose-free phenotype [115]. The loss-of-function of the *StGBSSI* protein resulted in the generation of tetra-allelic mutants with impaired amylose biosynthesis by targeting two loci encoding the StGBSSI enzyme. Using a cytidine base editor (CBE), DNA substitution was efficiently and precisely induced at the *KTGGL*-encoding locus, resulting in discrete variation in the amino acid sequence and the generation of a loss-of-function allele [116]. The complete knockout of *StGBSS* enzyme activity was confirmed in four-allele mutant lines via phenotypic studies of starch [35,36]. Similar research by Toinga-Villafuerte et al. showed mutations in all four *GBSS* alleles and the complete elimination of amylose from the tubers in one event (T2-7). Starch viscosity profiles of tubers from six different knockout events were determined, and the values reflected the amylopectin/amylose ratio [117]. A significantly increased editing efficiency in the granule-associated starch synthase gene at the protoplast level was obtained by replacing the *Arabidopsis* U6 promoter driving the expression of the CRISPR component with endogenous potato U6 promoters [118]. Previously, Kusano et al. applied the dMac3 translational enhancer to a Cas9 expression system with multiple gRNA genes. The efficiency of targeted mutagenesis was significantly increased following the use of dMac3-driven Cas9. The mutants were found to have low amylose content in their tubers [119]. Therefore, the *StSSR2* and *StGBSS* genes can be successfully edited to reduce SGA toxicity and eliminate amylose from potato tubers, respectively. Many types of starch branching enzymes (SBEs) have been found in potatoes. Among them, *SBE1*, *SBE2*, and *SBE3* are known as the major enzymes involved in amylopectin biosynthesis. Non-DNA GE was used to induce mutations in one or two branching enzyme (*SBE*) genes in tetraploid potatoes to develop starch with an increased amylose ratio and extended amylopectin chains. Using the RNP transfection of potato protoplasts, mutation rates of up to 72% were achieved. The large mutation variations were grouped into three types: Group 1 lines, with all *StSBE1* alleles; Group 2 lines, with all *StSBE1* alleles plus two to three *StSBE2* alleles; and Group 3 lines, with all alleles of both genes. Starch from Group 3 lines was found to be essentially free of amylopectin, showing no detectable branching and a chain length (CL) distribution that indicated the absence of both the dominant amylopectin fraction and the shortest amylose chains. Interestingly, the starch was still able to form granules with a low-ordered crystalline arrangement. Starch derived from Group 2 lines exhibited an increased CL, characterized by a higher amount of intermediate-sized chains and a modified granule phenotype, yet maintained a crystalline structure in the granules similar to that of wild type starch. Minor variations in CL were also observed for Group 1 starches when analyzed at a higher resolution [120]. Significant reductions in *StSBE1* and *StSBE2* reduced amylopectin branching during granule growth, whereas reductions in *StSBE2* alone primarily affected the number of starch granule initiations [121,122]. Takeuchi et al. generated mutants of the potato *StSBE3* gene using dMac3-driven CRISPR-Cas9. Mutations in the *StSBE3* gene were detected in 89 of 126 potato plant transformants. Western blot analysis revealed defective *StSBE3* production in the mutant tubers, leading the authors to suggest that these transformants were *StSBE3* loss-of-function mutants [123]. The results indicate that mutagenesis of *StSBE* genes has the potential to generate novel and potentially valuable starch properties without integrating foreign DNA into the genome. The *ALS* gene encodes an enzyme that catalyzes the initial step of the branched-chain amino acid biosynthetic pathway. Veillet et al. targeted the *ALS* gene in tomato and potato using CBE. They edited the target cytidine bases and produced edited but transgene-free plants in the first generation [124].

Anthocyanins are a group of plant pigments from the flavonoid class that impart red, blue, and purple hues to plants and have beneficial properties, such as antioxidant activity. In addition, they play an important role in plant responses to abiotic and biotic stresses. The *StMYB* genes of the R2R3 MYB transcription factor in potato play an important role in regulating anthocyanin accumulation and tuber flesh color. The annotation of the reference genome DMv8.1 listed the *Pf* locus containing the following *R2R3 MYB TF* genes: *StMYB120*, *StMYB130*, *StMYB170*, *StMYB180*, *StMYB200*, *StMYB210*, *StMYB240*, and *StMYB270*. The editing of these genes showed that *StMYB200* and *StMYB210* were involved in the biosynthesis of anthocyanins in the tuber flesh. Anthocyanins were not synthesized when the *StMYB210* gene was knocked out [125].

Vacuolar invertase (Vinv) is one of the enzymes involved in cold-induced sweetening, responsible for the conversion of sucrose to glucose and fructose [126,127,128]. Vinv transcription is significantly upregulated under cold conditions, but it is downregulated under normal temperatures. Tubers from *StVinv* knockout lines had undetectable levels of reducing sugars and low acrylamide levels and produced light-colored potatoes. Ly et al. used CRISPR-Cas9 to edit the *Vinv* and *asparagine synthetase 1* (*AS1*) genes of Atlantic and Desiree varieties, to reduce the accumulation of reducing sugars and asparagine production after cold storage. The Atlantic and Desiree tubers had reduced fructose and glucose concentrations after cold storage. The Desiree potato chips were also lighter in color and contained 85% less acrylamide [38]. Similar research on editing the *StVinv* gene demonstrated the reduced expression and lower enzymatic activity of *StVinv*, resulting in reduced sugar formation during cold storage after the harvest and sweetening of potatoes [29]. In addition, other scientists have shown that the knockout of the *StVinv* gene can improve root development [129] and preserve the quality of potato tubers during cold storage [130]. These results indicate that multiplexed CRISPR-Cas9 technology can generate improved potato varieties for healthier processed potato products.

Suberin is a lipid biopolymer that is an important structural component of plant tissues, acting as a barrier to diffusion and transpiration, especially in the root endoderm and periderm of potato tubers. To assess the importance of suberin in this process, loss-of-function mutants of the suberin transporter gene *StABCG1* were generated. GE resulted in decreased wound suberin production, accompanied by significant transcriptomic, metabolomic changes, and tissue browning [131].

**Table 4:** Using CRISPR-Cas technology for potato tuber quality improvement.

Target Gene	Delivery Method	Type of Editing	Improvement	References
*StSN2*	*Agrobacterium*- mediated transformation	Knockout	Tuber formation	[95]
*StCDF1* promoter	*Agrobacterium*- mediated transformation	Knockout	Potato tuberization	[96]
*StSP6A*	*Agrobacterium*- mediated transformation	Knockout	Induction and formation of potato tubers	[97]
*StIT1*	*Agrobacterium*- mediated transformation	Knockout	Tuber initiation	[99]
*StF3H*	*Agrobacterium*- mediated transformation	Knockout	Skin color change	[98]
*StPPO*	*Agrobacterium*- mediated transformation	Knockout	Reduced enzymatic browning	[104]
*StPPO1*, *StPPO2*	*Agrobactierum*- and *geminivirus*-based transformation	Knockout	Reduced enzymatic browning	[41]
*StPPO2*	Protoplast transfection with RNPs	Knockout	Reduced enzymatic browning	[101,102]
*StFtsZ1*	*Agrobacterium*- mediated transformation	Knockout	Increase in starch granule size	[109]
*StSS5*	*Agrobacterium*- mediated transformation	Knockout	Starch granule formation and tuber development	[110]
*StSS6*	*Agrobacterium*- mediated transformation	Knockout	Starch structure	[111]
*StGWD1*	*Agrobacterium*- mediated transformation	Knockout	Amylose content	[113]
*StSSR2*	*Agrobacterium*- mediated transformation	Knockout	Decreasing level of SGA	[30]
*St16DOX*	*Agrobacterium*- mediated transformation	Knockout	Decreasing level of SGA	[114]
*StGBSS*	*Agrobacterium*- mediated transformation	Knockout	Amylose content decrease	[115]
*StGBSS*	PEG-mediated protoplast transfection	Knockout	Amylose content decrease	[36,116]
*StGBSS*	PEG-mediated protoplast transfection	Knockout	Amylose content decrease	[35]
*StGBSSI*	*Agrobacterium*- mediated transformation	Knockout	Amylose content decrease	[117]
*StGBSSI*	*Agrobacterium*- mediated transformation	Knockout	Amylose content decrease	[118]
*StGBSSI*	*Agrobacterium*- mediated transformation	Base edit	Amylose content decrease	[119]
*StSBE1*, *StSBE2*	*Agrobacterium*- mediated transformation	Knockout	Amylose content decrease	[120]
*StSBE1*, *StSBE2*	*Agrobacterium*-mediated transformation and by PEG-mediated delivery into protoplasts	Knockout	Decrease in branching frequency of amylopectin	[121,122]
*StSBE3*	*Agrobacterium*- mediated transformation	Knockout	Amylose/amylopectin content decrease	[123]
*StMYB210*	*Agrobacterium*- mediated transformation	Knockout	Regulation of anthocyanin accumulation in tuber flesh	[125]
*StVinv*, *StAS1*	RNP-particle bombardment and *Agrobacterium*- mediated transformation	Knockout	Reduced browning and acrylamide	[38]
*StVinv*	*Agrobacterium*- mediated transformation	Knockout	Optimize level of sugars	[29]
*StVinv*	*Agrobacterium*- mediated transformation	Knockout	Optimize level of sugars	[130]
*StVinv*	*Agrobacterium*- mediated transformation	Knockout	Root development	[129]
*StInvVac*	Protoplast transfection	Knockout	Long-term cold storage and bruising resistance	[126]
*StABCG1*	*Agrobacterium*- mediated transformation	Knockout	Reduced suberin formation	[131]

Tuber quality plays a key role in determining the market value and commercial attributes of potatoes. In general, crop quality is determined by attributes such as tuber shape, skin and flesh color, starch content, and enzymatic browning. GE techniques, such as CRISPR, can be used to improve potato tuber quality. Numerous studies have been conducted on GE using CRISPR-Cas to alter skin color [98], carotenoid content [99], enzymatic browning [38,41,100,101,102,104,126], and starch structure [109,111], as well as to reduce SGA levels [30,114,132], optimize amylose and amylopectin content [35,36,115,116,117,118,119,120,121,122,123], and improve storage qualities [29,38,126,130]. The CRISPR-Cas system has already achieved significant success in improving potato tuber quality, and it is expected to inevitably play an important role in improving potato quality in the future.

## 6. Challenges and Future Perspectives

Currently, GE in crops is advancing at a much faster rate than in other fields. CRISPR-Cas technology is being actively used in potato production to improve the genetic properties of potatoes. However, there are some serious challenges that potato production faces.

Modern GE techniques, such as CRISPR-Cas9, are promising methods for creating disease-resistant varieties. The introduction of disease resistance genes is not an optimal method as pathogens quickly adapt to them, resulting in resistance being lost in a short time. Given the functional conservation of *S* genes, it is possible to create resistant lines for robust and broad disease resistance [61]. Using CRISPR-Cas9, various studies have been conducted on potatoes in recent years that involve the site-directed mutagenesis of *S* genes [62,69,75,76]. Hereafter, future research should focus on enhancing the accuracy and efficiency of CRISPR-Cas9 technology to develop resistant potato lines.

The use of pesticides is undoubtedly one of the main negative factors affecting the environment. Pesticides can lead to disturbances in ecosystems, a decrease in biodiversity, and food contamination [133]. Therefore, organic farming has recently received increased attention due to growing concerns about health and environmental issues. Organic potato farming is an approach that eliminates the use of synthetic fertilizers and pesticides. Instead, organic farmers use naturally occurring methods to maintain the health of the population, soil, and plants. GE via CRISPR-Cas can help organic farming by improving plant characteristics, such as pest and disease resistance, environmental adaptation, and increased yields, which ultimately reduces the need for pesticides and other chemicals. To date, research has been conducted on using GE to impart resistance to fungal and viral diseases in potatoes (Table 2). Researchers have developed lines resistant to weeds [134,135], bacterial diseases [73], and herbicides [86]. Therefore, future studies may apply CRISPR-Cas technologies to the development of potato varieties and lines more suitable for organic farming. However, CRISPR-Cas GE is categorized as a biotechnology, and its use is therefore prohibited in organic farming under regulations established by the United States Department of Agriculture (USDA) [136] and the European Union [137]. The authors of this review consider the use of pesticides to pose a more urgent concern, given their detrimental impacts on the environment and human health. Consequently, for future applications, GE crops modified via CRISPR-Cas9 have been proposed for use in organic farming [138]. The GE methods can be made to exclude any foreign DNA and vectors in the end products, making them indistinguishable from those produced through traditional breeding methods.

The development of varieties resistant to common diseases is a key aspect of plant breeding and is aimed at increasing crop productivity and resistance to harmful environmental influences, such as diseases and pests. This challenge is especially relevant for developing countries that rely on imported seed potatoes for production and processing. Moreover, there is a particular need to develop plant breeding in developing countries to develop varieties adapted to local conditions. This will increase self-sufficiency via potatoes and improve food security in developing countries [139]. Therefore, CRISPR-Cas technology shows great potential for developing non-transgenic potatoes with edited, desired genes and improved traits [108,126]. However, for commercialization, the political and technical limitations of GE crops need to be addressed. Regulation and policy for GE crops are controversial, as different countries have different rulings, and, in many countries, the rules for GE plants are subordinate to the rules on genetically modified organisms (GMOs). The European Union and New Zealand have strict rulings for GE crops [137,138,140,141]. In contrast, in the United States, Argentina, Brazil, Chile, and Colombia, the rulings are that the final product must not contain exogenous DNA [142,143,144,145,146]. In China, the most recent regulations simplify the approval process for GE crops that do not include foreign genes; however, a case-by-case assessment system is still applied regarding possible environmental and human health risks [147]. Crops containing foreign genes are largely still subject to GMO standards, and legislation governing the commercialization of modified crops remains largely unchanged. However, in 2025, China began commercializing GE for five crops, including wheat, corn, and soybean varieties [148]. They represent a significant step in the country’s approach to agricultural biotechnology and food security, as the country continues its efforts to reduce import dependence and ensure food security. Overall, the regulatory framework for GE crops is currently under development and varies by country and specific application. Common approaches include risk assessment, labeling, tracking, and discussions of public acceptance. Therefore, a global, tailored regulatory framework for GE crops needs to be established.

One of the limitations of GE can be seen in gene knock-in, a technique that enables precise DNA sequence insertion at a specific location via the HDR pathway following a DSB. This process requires introducing donor DNA with the desired sequence alongside a single-strand nick (SSN) or other means to trigger HDR. However, HDR’s low frequency in plant cells makes gene knock-in relatively inefficient and underutilized compared to other GE methods, such as gene knockout. Nevertheless, a novel strategy utilizing the CRISPR/Cas9 RNP complex for HDR-mediated knock-in in Petunia protoplasts has shown promise for successful gene integration [149]. Several other approaches have also been proposed for increasing knock-in efficiency, including HDR pathways, NHEJ- and microhomology-mediated end joining (MMEJ)-mediated knock-ins, and integrations into geminiviral vectors [26]. Future improvements in GE are expected to lead to greater efficiency and reliability of gene knock-in, opening new possibilities for agriculture.

The most common method of Cas9 cassette expression in plants is *Agrobacterium*-mediated transformation using a plasmid containing the Cas9 protein and sgRNA sequence [150]. After transformation, *Agrobacterium* integrates the CRISPR/Cas9 cassette into the plant genome, which can lead to various undesirable consequences. These include constitutive expression of Cas9 proteins and off-target effects [151]; also, the presence of the Cas9 gene in the plant genome can raise ethical issues. Most engineered plasmids use separate promoters for Cas9 and gRNA, the 35S promoter derived from cauliflower mosaic virus, or other codon-optimized analogs for Cas9 and U3/U6 promoters for gRNA synthesis [152], which limits the maximum number of expressed gRNAs [153]. The problems encountered in *Agrobacterium*-mediated transformation can be reduced by removing the transgene after successful integration or by using other DNA-free transformation methods, such as RNPs consisting of Cas9 protein and sgRNA, or viruses. RNPs have the advantage of low off-target effects due to their rapid degradation after integration [154]. The RNP delivery method faces difficulties due to the plant cell wall, but these can be mitigated by isolating protoplasts and transfecting with PEG [155]. This method has been widely used in potato [35]. Another method to deliver the Cas9 cassette without transgenes is the use of viral vectors. Compared to RNPs, the delivery of viral vectors does not necessitate protoplast isolation and therefore does not require tissue regeneration, a labor-intensive and time-consuming process. Viral vectors enable the transient expression of the Cas9 cassette and, when systemically propagated, lead to efficient mutations in all plant tissues [156]. One disadvantage of RNA viruses is low cargo capacity and difficulty in incorporating the full CRISPR cassette into their genome. However, delivery of the full CRISPR cassette using viral vectors has been demonstrated in other plant cultures [157].

In potato breeding, priority is given to breeding methods that ensure the rapid production of commercial tubers from TPS, as this provides a healthy crop. Seed propagation using TPSs in breeding enables the production of large volumes of planting material with high genetic diversity and, importantly, a greater likelihood of avoiding vegetatively transmitted diseases [158]. Diploid species are widespread (>70%), but they are self-incompatible; therefore, inbred lines were not previously possible. For overcoming the self-incompatibility of diploid species, the *Sli* (*S*-locus inhibitor) gene was discovered, which suppresses self-incompatibility and creates self-compatible diploid lines [159]. GE CRISPR-Cas9 overcomes self-incompatibility in diploid potato to develop self-compatible potato lines. Thus, the genomic engineering of diploid *F1* potato hybrids is now feasible in potato breeding for the purpose of obtaining TPS. De novo domestication of wild potatoes is a possible way of introducing new trait donors for expanding the pool of desirable genes and agronomic traits in the potato crop—specifically, genes conferring resistance to biotic and abiotic stresses. Domestication via CRISPR-Cas genome editing has been experimentally performed on tomato [160]. Egorova et al. describe a three-stage strategy for domestication of wild potato for breeding purposes, where CRISPR-Cas can be utilized at every stage. Firstly, a set of candidate domestication genes must be found and experimentally validated, which may be performed via precise CRISPR-Cas genome editing to modify the potato genome(s) and observe its effects. Secondly, wild potato genotypes with comprehensive annotation of desirable and undesirable genes must be documented; again, CRISPR-Cas may be used to study the roles and effects of genes in wild potato, such as via gene knockouts. Finally, an effective platform for genetic transformation and in vitro regeneration of modified wild potato must be established. Proofs-of-concept for separate stages of this strategy were described by Egorova et al.; however, a successful attempt for wild potato has not been documented yet [161]. Among the problems is insufficient documentation of wild potato genotype information and genebanks, difficulty of prediction or screening for certain desirable traits, such as disease resistance, and the need for the development of a universal platform for genetic transformation and regeneration. Overall, the potential for CRISPR-Cas applications in potato hybrid breeding, interspecies crossing, self-compatibility, and de novo speciation accelerates the process of potato breeding.

The need for import substitution is particularly acute for potato varieties destined for deep processing, specifically those used to produce chips and French fries. At present, developing countries require their own varieties and seeds for growing potatoes for processing. In the long term, CRISPR-Cas technology is likely to have a significant impact on the future of potato breeding, especially for creating varieties suitable for efficient processing.

Currently, breeders are working to re-conceptualize the potato as a diploid crop to accelerate progress in understanding the genetics of complex traits such as yield, quality, and drought tolerance [162]. GE, combined with inbred diploid line development, will represent a monumental shift in the potential for genetic improvement, opening the door to a more effective potato-breeding pipeline [163]. The transition to diploid potatoes enables the development of hybrids based on selected inbred lines, which can improve various agronomic traits, such as resistance to diseases and abiotic stresses, such as drought, salinity, and low and high temperatures.

## 7. Conclusions

Currently, the fastest and most efficient method for obtaining plants with desired traits is GE via CRISPR-Cas. This technology is used in plant breeding to enhance resistance to diseases and abiotic stresses, improve tuber quality, and increase the efficiency of breeding programs by enabling the precise and rapid modification of the plant genome. Despite advances in CRISPR-Cas GE, most work on crop improvement remains at the stage of elucidating genomic function and regulatory mechanisms. Additionally, public concerns and strict country-specific policies regarding gene editing methods are another obstacle to the introduction of precision potato breeding. Despite these remaining challenges, CRISPR-Cas technology is expected to be more widely adopted in the future and will inevitably play an important role in enhancing potato quality and yield.

## Figures and Tables

**Table 1:** Using CRISPR-Cas technology for the breeding process in potato.

Target Gene	Delivery Method	Type of Editing	Improvement	References
*StS-RNase*	*Agrobacterium*- mediated transformation	Knockout	Pollen tube growth	[45,46,47,48]
*StSli*	*Agrobacterium*- mediated transformation	Knockout	Overcoming self-pollen rejection	[46]
*StTaALS*	*Agrobacterium*- mediated transformation	Base editing	Fundamental research	[47]
*StALS1*	*Agrobacterium*- mediated transformation	Knockout	Herbicide resistance	[20]
*StALS1*	*Agrobacterium*- and *geminivirus*-mediated GE	Knockout	Herbicide tolerance	[48]
*StScINVINH1*	*Agrobacterium*- mediated transformation	Knockout	Callus induction and regeneration	[49]
*StPDS*	*Agrobacterium tumefaciens*-based transformation	Knockout	Broadens the number of genotypes for transformation and reduces chimerism	[50]
*StDwarf*, *StEr*	*Agrobacterium*- mediated transformation	Knockout	Plant growth	[51]

**Table 2:** Using CRISPR-Cas technology for potato disease resistance.

Target Gene	Delivery Method	Type of Editing	Improvement	References
*StNRL1*	*Agrobacterium*- mediated transformation	Knockout	Late blight resistance	[54]
*StDND1*, *StCHL1*, *StDMR6-1*	*Agrobacterium*- mediated transformation	Knockout	Late blight resistance	[62]
*StDMR6-1*	*Agrobacterium*- mediated transformation	Knockout	Late blight, early blight, and common scab	[66]
*StPM1*	*Agrobacterium*- mediated transformation	Knockout	Late blight resistance	[67]
*StCCoAOMT*	*Agrobacterium*- mediated transformation	Knockout	Late blight resistance	[68]
*StERF3*	*Agrobacterium*- mediated transformation	Knockout	Late blight resistance	[69]
*StDMP2*	*Agrobacterium*- mediated transformation	Knockout	Late blight resistance	[70]
*StMC7*	*Agrobacterium*-mediated transformation	Knockout	Late blight resistance	[71]
*StSR4*	RNP-mediated CRISPR/Cas9 GE	Knockout	Late blight resistance	[72]
*StNPR3*	*Agrobacterium*-mediated transformation	Knockout	Resistance to Clso	[73]
*StCoilin* gene	RNP-mediated CRISPR/Cas9 GE	Knockout	Potato virus Y	[74]
*SteIF4E*	*Agrobacterium*-mediated transformation	Knockout	Potato virus Y	[75]
*SteIF4E1*	Protoplast transfection	Knockout	Potato virus Y	[76]
*StP3*, *StCI*, *StNib*, *StCP*	*Agrobacterium*-mediated transformation	Virus RNA targeting	Potato virus Y	[77]
*StPI*, *StHC-Pro*, *StP3*, *StCl1*, *StCl2*, and *StVPg*	*Agrobacterium*-mediated transformation	Virus RNA targeting	Potato virus Y	[78]
*StCP* gene	*Agrobacterium*-mediated transformation	Virus RNA targeting	Multiplex virus resistance PVY, PVS, PVX, or PLRV	[79]

**Table 3:** Using CRISPR-Cas technology for abiotic stress tolerance in potatoes.

Target Gene	Delivery Method	Type of Editing	Improvement	References
*StDMR6-1*	*Agrobacterium*- mediated transformation	Knockout	Drought and salt tolerance	[66]
*StDRO2*	*Agrobacterium*- mediated transformation	Nonsense mutation	Drought tolerance	[92]
*StAOX*	*Agrobacterium*- mediated transformation	Knockout	High-light tolerance	[93]
*StMYB44*	*Agrobacterium*- mediated transformation	Knockout	Pi transport regulation	[94]
*StbHLH47*	*Agrobacterium*- mediated transformation	Knockout	Decreased ferric chelate reductase (FCR) activity	[91]

## Data Availability

No new data were created or analyzed in this study. Data sharing is not applicable to this article.

## Abbreviations

The following abbreviations are used in this manuscript:

CRISPR	clustered regularly interspaced short palindromic repeats
Cas	CRISPR-associated protein
MAS	marker-assisted selection
GE	gene editing
ZFN	zinc finger nuclease
TALEN	transcription activator-like effector nuclease
(s)gRNA	(Single) Guide RNA (Ribonucleic Acid)
CRISPRa	CRISPR-mediated transcriptional activation
CRISPRi	CRISPR interference
BE	base editing
PE	prime editing
DSB	double-strand break
HR	homologous recombination
NHEJ	non-homologous end joining
HDR	homology-directed repair
PEG-mediated	polyethylene glycol-mediated
RNAi	RNA interference
StNRL1	*Solanum tuberosum* NPH3/RPT2-like1
MLO	Mildew Locus O
SWEET	Sugars Will Eventually be Exported Transporter
*StDMR6*	*S. tuberosum* downy mildew resistance 6
*StbHLH47*	*S. tuberosum* basic helix–loop–helix 47
*StDRO2*	*S. tuberosum* deeper rooting 2
RSA	root system architecture
*StLike3*	*S. tuberosum* FMO GS-OX-Like3
*StDND1*	*S. tuberosum* defense no death 1
*StCHL1*	*S. tuberosum* CIB1/HBI1-like1
*StPM1*	*S. tuberosum* PLASMA MEMBRANE PROTEIN 1
AWPM-19	ABA-induced wheat plasma membrane polypeptide-19
*StCCoAOMT*	*S. tuberosum* Caffeoyl-CoA O-methyltransferase
*StERFs*	*S. tuberosum* ethylene response factors
*StDMP2*	*S. tuberosum* DOMAIN OF UNKNOWN FUNCTION 679 membrane protein 2
ER	endoplasmic reticulum
*StPRRs*	*S. tuberosum* pattern recognition receptors
*StNDR1*	*S. tuberosum* non-race-specific disease resistance 1
MC	metacaspase
PCD	programmed cell death
*StMC7*	*S. tuberosum* metacaspase 7
SR1	Signal Response 1
*StSR4*	*S. tuberosum Signal Responsive 4*
CAMTA3	calmodulin (CaM)-binding transcription activator 3
Clso	*Candidatus Liberibacter solanacearum*
*StNPR(3)*	*S. tuberosum* non-expressor of pathogenesis-related (3)
PVY	potato virus Y
*SteIF*	*S. tuberosum* eukaryotic translation initiation factor
nCBP	New Cap-Binding Protein
Cas13a	CRISPR-associated protein “a”
NIb	Nuclear Inclusion protein b
CP	Coat Protein
Cas13d	CRISPR-associated protein “d”
PTG	Polycistronic tRNA-gRNA
LshCas13a	*Leptotrichia shahii* Cas13a
EPSPS	5-enolpyruvylshikimate-3-phosphate synthase
GVR	geminivirus replicon
*StALS*	*S. tuberosum* Acetolactate Synthase
PVS	potato virus S
PVX	potato virus X
PLRV	potato leafroll virus
*StAOX*	*S. tuberosum* alternative oxidase
ROS	reactive oxygen species
Pi	phosphate
RNA-Seq	RNA sequencing
*StMYB44*	*S. tuberosum* MYB 44 transcription factor in *S. tuberosum*
*StVinv, StInvVac*	*S. tuberosum* vacuolar invertase
*StSN2*	*S. tuberosum* Snakin-2
ABA	abscisic acid
STP1	Sugar Transporter Protein 1
*StCDF1*	*S. tuberosum* cycling dof factor 1
*StSP6A*	*S. tuberosum self-pruning 6A*
*StPHO1*	*S. tuberosum* phosphate transporter 1
S-RNAse	S-locus RNAse
SLF	S-locus F-box
Sli	S-locus inhibitor
SaCas9	*Staphylococcus aureus* CRISPR-Cas9
GBSS	granule-bound starch synthase
*StGBSS*	*S. tuberosum* granule-bound starch synthase
*StGBSS(I)*	*S. tuberosum* granule-bound starch synthase (I)
INVINH1	invertase inhibitor 1
*StPDS*	*S. tuberosum* phytoendesaturase
*StER*	*S. tuberosum Erecta*
B33	also named Patatin class I promoter
Suc, SUT	sucrose transporter
AmA1	Amaranth Albumin 1
*StF3H*	*S. tuberosum* Flavanone 3-hydroxylase
*StPPO*	*S. tuberosum* Polyphenol Oxidase
pGEF(-U)	Fluorescent Editor Geminivirus-Based Plasmid (Universal)
GTPase	Guanosine Triphosphate hydrolase
*StFtsZ1*	*S. tuberosum* Filamentous temperature sensitive 1
SNP	Single Nucleotide Polymorphism
SGAs	steroidal glycoalkaloids
StSSR2	*S. tuberosum* sterol side chain reductase 2
2OGD	2-oxoglutarate-dependent dioxygenase
*St16DOX*	*S. tuberosum* 16-hydroxylase
*StSS5*	*S. tuberosum* starch synthease 5
*StSS6*	*S. tuberosum* starch synthease 6
*StGWD1*	*S. tuberosum* α-glucan water dikinase 1
*StMYB210*	*S. tuberosum R2R3 MYB transcription factor genes*
pre-tRNA	precursor of transfer RNA
CBE	cytidine base editor
*StSBE*	*S. tuberosum* Starch Branching Enzyme
RNP	ribonucleoprotein
CL	chain length
*StIAA*	*S. tuberosum* auxin/indole-3-acetic acid
*StABCG1*	*S. tuberosum* suberin transporter
*StAS1*	*S. tuberosum* asparagine synthetase 1
TPS	true potato seed
IR	Intergenic Region
SSN	single-strand nick
